# Chronic Alcohol Exposure Induces Aberrant Mitochondrial Morphology and Inhibits Respiratory Capacity in the Medial Prefrontal Cortex of Mice

**DOI:** 10.3389/fnins.2020.561173

**Published:** 2020-10-22

**Authors:** Pei Shang, Daniel Lindberg, Phillip Starski, Lee Peyton, Sa-Ik Hong, Sun Choi, Doo-Sup Choi

**Affiliations:** ^1^Department of Molecular Pharmacology and Experimental Therapeutics, Mayo Clinic College of Medicine and Science, Rochester, MN, United States; ^2^Department of Neurology, First Hospital of Jilin University, Changchun, China; ^3^Neuroscience Program, Mayo Clinic College of Medicine and Science, Rochester, MN, United States; ^4^Department of Psychiatry and Psychology, Mayo Clinic College of Medicine and Science, Rochester, MN, United States

**Keywords:** alcohol use disorder, fission, fusion, medial prefrontal cortex, mitochondria, morphology, respiratory capacity

## Abstract

Alcohol use disorder (AUD) is characterized as a chronic, relapsing disease with a pattern of excessive drinking despite negative consequences to an individual’s life. Severe chronic alcohol use impairs the function of the medial prefrontal cortex (mPFC), which contributes to alcohol-induced cognitive and executive dysfunction. The mPFC contains more mitochondria compared to other cortical areas, which suggests mitochondrial damage may occur in AUD and trigger subsequent behavior change. Here, we identified morphological and functional changes in mitochondria in the mPFC in C57BL6/J mice after 8 h of withdrawal from chronic intermittent alcohol (CIA) exposure. Three-dimensional serial block-face scanning electron microscopy (SBFSEM) reconstruction revealed that CIA exposure elongated mPFC mitochondria and formed mitochondria-on-a-string (MOAS). Furthermore, alcohol significantly affected mitochondrial bioenergetics, including oxidative phosphorylation and electron transport, with inhibited aerobic respiration in mPFC mitochondria after CIA exposure. We also found decreased expression of fusion (mitofusin 2, Mfn2) and increased fission (mitochondrial fission 1 protein, Fis1) proteins in the mPFC of alcohol-treated mice. In sum, our study suggests that CIA exposure impairs mitochondrial dynamics and function in the mPFC.

## Introduction

Alcohol use disorder (AUD) is ranked among the most prevalent mental disorders disproportionately affecting men (8.6% men vs. 1.7% women in 2016) (Rehm and Shield, 2019). Impaired control over alcohol consumption leads to an escalating pattern of alcohol use, which is a mainstay clinical feature in patients with such disorders, despite the patients’ recognition of the detriments to health, relationships, and social activities (Carvalho et al., 2019). Additionally, AUD is associated with cognitive, emotional, and behavioral impairments (Oscar-Berman and Marinkovic, 2007). Despite numerous studies on the direct and indirect molecular targets of alcohol’s action, the need to develop novel therapeutic strategies to target brain circuits or behavior remains (Abrahao et al., 2017).

In the central nervous system (CNS), previous studies demonstrated that chronic alcohol exposure induces structural and functional changes in several brain regions. For example, functional MRI (fMRI) from abstinent patients with AUD exhibits alterations in brain regions associated with cognitive function and spatial and working memory (Tapert et al., 2001; Desmond et al., 2003), vigilance (Tapert et al., 2001), and proactive interference (De Rosa et al., 2004). Several brain regions, including the neocortex (especially frontal lobes), limbic system, and cerebellum, are known to be more vulnerable to the effects of alcohol (Moselhy et al., 2001; Oscar-Berman and Marinkovic, 2003; Sullivan, 2003). In line with these findings, PET, perfusion-weighted MRI, and SPECT also showed a significant decrease in cortical metabolism (Dao-Castellana et al., 1998; Gansler et al., 2000; Clark et al., 2007). Structural neuroimaging and neuropathological studies further supported that the frontal lobes are susceptible to alcohol-induced brain damage (Harper and Matsumoto, 2005; Sullivan and Pfefferbaum, 2005). Notably, the increased activities in a fronto–parieto–cerebellar network in patients with AUD might compensate for the deficiency in dorsolateral prefrontal and parietal circuits, aiding in the facilitation of accurate behavioral performance (Sullivan and Pfefferbaum, 2005). Moreover, projections from mPFC to subcortical sites, including the extended amygdala and nucleus accumbens, were disrupted in AUDs, causing abnormalities in impulsivity and associative learning (Klenowski, 2018). According to our previous study, chronic intermittent alcohol (CIA) exposure triggers abnormal impulse and reward-seeking behaviors, which are associated with the mPFC and underlying changes in proteomic profiles (Starski et al., 2019a).

Since recurrent firing to sustain working memory function requires large amounts of energy, the mPFC contains more mitochondria than other cortical areas (Arnsten et al., 2012). Mitochondria are essential in energy production, metabolic pathways, signaling transduction, lipid biogenesis, and apoptosis, relying on around 1000 resident proteins (Araiso et al., 2019). The size of mitochondria varies from 0.5 to 10 μm depending on host cell types and specific intracellular localization. The basic three-dimensional architecture of mitochondria consists of an outer membrane, cristae formed by inner membrane, an electron transport chain located at the inner membrane, intermembrane space, and a matrix (Trushina, 2016). In the CNS, mitochondria are trafficked throughout the axons and dendrites in neurons and rely on the balance between fission and fusion events for optimal function (Li et al., 2004; Chen and Chan, 2009). Membrane fission and fusion can convert mitochondria from an integrated mitochondrial reticular network to a fragmented network or *vice versa*, accompanied by the dynamic interchanges of the sharing of inner and outer membrane constituents and matrix contents (Brown et al., 2001; Sancho-Tello et al., 2008). Mitochondria on a string (MOAS) represent multiple teardrop-shaped mitochondria connected by a thin double membrane extending up to 5 μm. The elongated interconnected mitochondria represent an unknown mitochondrial fission arrest phenotype that was first described in hippocampal tissue from human and mouse models of Alzheimer’s disease (AD) (Zhang et al., 2016).

Mitochondrial dynamics sustain the cellular integrity and manage the dispersal and disposal of abnormal constituents in the mitochondrial network (Kim et al., 2007; Youle and Narendra, 2011). Previous studies have shown that alcohol promotes cell apoptosis, alters mitochondrial dynamics, and affects mitochondrial morphology in a concentration-dependent manner (Kitagaki et al., 2007; Das et al., 2012). Mitochondria were misshapen and enlarged, accompanied by metabolic dysfunction in liver and human retinal pigment epithelial cells after alcohol exposure (Suen et al., 2008; Flores-Bellver et al., 2014). Mitochondrial fission and fusion events are mainly regulated by dynamin-related protein 1 (Drp1), fission-1 (Fis1), optic atrophy 1 (OPA1), and mitofusin-2 (Mfn-2) proteins (Santel and Fuller, 2001; Lee et al., 2004; Suen et al., 2008). Drp1 levels are significantly increased after alcohol treatment in a concentration-dependent manner in human retinal pigment epithelial cell lines, promoting fission (Bonet-Ponce et al., 2015). However, a gap of knowledge remains pertaining to dynamic mitochondrial changes *in vivo*. Even fewer studies have combined the morphology alteration with the mitochondrial functional transition. Therefore, in this study, we identified the three-dimensional morphological alteration of MOAS in the mPFC of mice after CIA exposure. We also correlated the MOAS phenomenon with fusion and fission dynamics and elucidated functional changes in isolated mitochondria.

## Materials and Methods

### Animals

Male C57BL/6J mice aged 6 weeks were purchased from Jackson Laboratories Inc. (Bar Harbor, ME, United States). Mice were group-housed in standard Plexiglas cages with *ad libitum* access to food and water immediately after arrival. Mice were maintained on a 12 h:12 h light- and-dark cycle (dark cycle from 1800 to 0600). Animal care and handling procedures were approved by the Mayo Clinic Institutional Animal Care and Use Committees in accordance with NIH guidelines.

### Chronic Intermittent Alcohol Exposure

Alcohol vapor or air was delivered in Plexiglas inhalation chambers, as previously described (Griffin et al., 2009). We have used the vapor chamber system previously to successfully maintain stable blood alcohol concentration (BAC) in mice (Hong et al., 2019; Starski et al., 2019a). Mice were exposed to alcohol vapor or room air using vapor administration chambers for 16 h from 1600 to 0900, followed by 8 h of room air in their home cages. In this study, mice were not given pyrazole, an alcohol dehydrogenase inhibitor that may affect the oxidative phosphorylation processes in mitochondria (Cederbaum and Rubin, 1974). This process was repeated for four consecutive days, followed by 3 days in their home cages with room air (withdrawal period). Before each 16-h exposure, mice in alcohol groups were administered a priming injection of 1.5 g/kg ethanol solution to maintain a stable level of intoxication during ethanol vapor exposure. Mice in air groups received an injection of 0.9% saline before being placed in air control chambers. After the last alcohol exposure, mice tail blood was collected immediately and centrifuged to extract the serum. BACs were measured by Analox GL5 multimetabolite analyzer (Analox Instruments, Stourbridge, United Kingdom) with the accompanying kits. The target maximum BAC is about 200 mg ethanol/dl of blood in the vapor chamber, as shown in our previous studies (Lindberg et al., 2019; Starski et al., 2019b). After four cycles of CIA and 8 h of withdrawal, mice were anesthetized with an intraperitoneal injection of ketamine/xylazine (ketamine 100 mg/kg, xylazine 8 mg/kg) and then euthanized via dislocation for Seahorse XF96 Analyzer and western blotting or prefusion for serial block-face scanning electron microscopy (SBFSEM) study.

### Serial Block-Face Scanning Electron Microscopy

Samples were stained and prepared for SBFSEM using an adapted protocol (Hua et al., 2015). Cardio perfusion was performed with 4% paraformaldehyde in anesthetized mice (IP injection of ketamine 100 mg/kg/xylazine 8 mg/kg), followed by whole brain removal. Brains were fixed by immersion in 2% glutaraldehyde + 2% paraformaldehyde in 0.15 M cacodylate buffer containing 2 mM calcium chloride for 24 h. Then coronal brain sections were prepared in a microtome (1-mm thickness), followed by the ACC being isolated using a 1-mm-diameter micro punch (Miltex, 33-31AA). Fixed samples were washed in 0.15 M cacodylate buffer and incubated at room temperature in 2% osmium tetroxide in 0.15 M cacodylate for 1.5 h. Without rinsing, samples were incubated in 2.5% potassium ferricyanide + 2% osmium tetroxide in 0.15 M cacodylate for another 1.5 h at room temperature. Following a rinse in nH_2_O, samples were incubated in 1% thiocarbohydrazide in H_2_O for 45 min at 50°C. After another rinse in nH_2_O, samples were incubated sequentially in 2% osmium tetroxide in H_2_O for 1.5 h at room temperature, 1% aqueous uranyl acetate overnight at 4°C, and 7% lead aspartate solution for 1 h at 50°C, with several rinses in nH_2_O between each reagent. Following dehydration through a series of ethanol and acetone rinses, samples were infiltrated and eventually embedded in polyepoxide resin Durcapan (EMS, Hatfield, PA, United States) and polymerized in a 60°C oven for 24 h. For placement into the SEM and subsequent imaging, embedded ACC tissue (1 mm^3^) was trimmed of any excess resin and mounted to 8-mm aluminum stubs using silver epoxy Epo-Tek (EMS, Hatfield, PA, United States). The mounted sample was then carefully trimmed to a 0.5-mm- × -0.5-mm- × -1-mm-tall tower using a diamond trimming knife (Diatome trimtool 45, EMS, Hatfield, PA, United States). The trimmed sample and entire stub were coated with gold palladium to assist in charge dissipation. The coated sample was then inserted into a VolumeScope serial block-face SEM (Thermo Fisher, Waltham, MA, United States) and allowed to acclimate in a high vacuum for 12 h prior to the start of imaging. High-resolution block-face images were obtained in a low-vacuum environment using beam energy of 1.8 kV with a current of 100 pA and a scanning dwell time of 2 μs and a 10-nm pixel size. A stack of approximately 350 block-face images was obtained while cutting the block at 50-nm increments. The image stack was then aligned and filtered using Amira software (Thermo Fisher, Waltham, MA, United States) with further analysis of mitochondria within dendrites performed using *Reconstruct* (Fiala, 2005).

### Three-Dimensional Serial Block-Face Scanning Electron Microscopy Reconstruction

For three-dimensional SBFSEM reconstruction, we collected a series of data by imaging the block surface, then removing an ultrathin section (50 nm) to reveal a new block face for imaging. Serial sections (300–350 slices per mouse) were obtained from the blocks. Images collected from serial sections were then stacked, aligned, and visualized using Amira software (Thermo Fisher, Waltham, MA, United States). Reconstructions of mitochondria within dendrites were performed on longitudinally sectioned neuropils that contained mitochondria in the alcohol group (*n* = 6) and the air group (*n* = 8). The tracked length, volume, area, and spaces between mitochondria were recorded. The average length of mitochondria was calculated via dividing tracked mitochondrial length by mitochondria amount. The average volume of mitochondria was calculated by dividing the total volume of tracked mitochondria by total mitochondrial length. The disconnectivity among mitochondria is reflected by the average space amount per mitochondrial length, calculated via dividing spaces in tracked mitochondria by the total length. The parameters were calculated in each tracked mitochondrion and then averaged in an individual mouse. MOAS is defined as teardrop-shaped mitochondria (approximate 0.5 μm in diameter) connected by a thin double membrane extending to 5 μm or longer (Zhang et al., 2016).

### Mito-Stress Assay and Seahorse XF96 Analyzer

We employed Seahorse procedures as previously described in isolated animal tissue (Jung and Metzger, 2015; Iuso et al., 2017). Seahorse XF96 Analyzer measures oxygen concentration and proton flux in wells of the Seahorse XF96 cell culture plate and calculates oxygen consumption rate (OCR) and extracellular acidification (ECAR) of intact cells or isolated mitochondria based on monitoring real-time aerobic respiration (Jung and Metzger, 2015; Iuso et al., 2017). Briefly, 8 h after removal from the last alcohol vapor exposure, anesthetized mice (IP injection of ketamine 100 mg/kg/xylazine 8 mg/kg; alcohol group *n* = 6 and air group *n* = 5) were euthanized via cervical dislocation, and the mPFC was dissected under a dissecting microscope. The mPFC was homogenized with the Potter-Elvehjem PTFE pestle and glass tube (Sigma-Aldrich, P7734-1EA) gently in mitochondria isolation buffer (210 mM d-Mannitol, 70 mM sucrose, 5 mM HEPES, 1 mM EGTA, and 0.5% (w/v) of fatty acid-free BSA in ultrapure H_2_O and adjust the pH to 7.2 with KOH at 37°C). Immediately following, mitochondria from mPFC were isolated by differential centrifugation as described (Iuso et al., 2017). The crude nuclei were removed from the lysate via centrifugation at 800 × *g* for 5 min at 4°C. The remaining supernatant was centrifuged at 8000 × *g* for 10 min at 4°C and the pellet was collected. Two washes were conducted for the pellet at 8000 × *g* for 10 min at 4°C. Mitochondria were then diluted with a mitochondria assay solution (MAS) with the substrates (220 mM mannitol, 70 mM sucrose, 10 mM KH_2_PO_4_, 5 mM MgCl_2_, 2 mM HEPES, 1 mM EGTA, and 0.2% (w/v) of fatty acid-free bovine serum albumin in ultrapure water, 10 mM pyruvate, 5 mM malate, PH to 7.2 with KOH at 37°C) to support complex I-associated respiration, and transferred into a Seahorse XF96 Analyzer (Seahorse Biosciences, North Billerica, MA, United States) cell culture plate with the same concentration (10 μg/well) measured with Bradford protein assay (Bio-Rad, Hercules, CA, United States). The plate was centrifuged (4°C, at 3000 × *g*, 20 min), and loaded into the Seahorse XF96 respirometer. Adenosine diphosphate (ADP) (40 mM) and Mito-stress drugs (20 μM oligomycin, 10 μM carbonyl cyanide-*p*-trifluoromethoxyphenylhydrazone (FCCP), and 10 μM rotenone/antimycin A diluted in 1X MAS buffer without substrate) were loaded on the dehydrated Seahorse XF96 Sensor Cartridge. Next, XF96 Analyzer was calibrated with calibrant buffer prior to injections as instructed by the manufacturer. OCR and ECAR were measured every 6 min for a total of 15 measurements. The OCR values were collected for a total of 15 measurements as P1 ∼ P15. OCR and ECAR curves were divided into basal level, State III, State IIIu, and State IVo. The calculation of each phase followed the previously described protocol (Iuso et al., 2017). The basal level represents the baseline respiration in the presence of substrates but without ADP and was calculated by the mean value of baseline [Average(P1 + P2 + P3)] subtracting background value α (non-mitochondrial O_2_ consumption) [Average(P13 + P14 + P15)]. State III represents phosphorylating respiration capacity relying on ATP-synthase calculated with the mean value of the P4 subtracting α. State IIIu represents adenosine triphosphate (ATP) uncoupler-stimulated maximal respiratory capacity (MRC) after adding FCCP reflects substrate oxidation, which is calculated by the mean value of the P10 subtracting α. State IVo represents non-phosphorylating, or proton conductance after oligomycin inhibited ATP synthase, and was calculated as the mean value after adding oligomycin [Average (P7 + P8 + P9)] subtracting α. Respiratory control ratio (RCR) was assessed with State III/State IVo or State IIIu/State IVo. The RCR value is 8.93 ± 0.1016 in the alcohol group and 8.282 ± 0.2115 in the air group (State III/State IVo) or 5.779 ± 0.3488 in the alcohol group and 5.868 ± 0.3314 in the air group (State IIIu/State IVo). The RCR values reflect the integrity of mitochondrial membrane and quality of prepared mitochondria to control heterogeneity among groups. Higher RCR (>4) represents normal function in isolated mitochondria.

### Western Blotting

For western blotting (WB) analysis, resuspended mPFC homogenates (alcohol group *n* = 4, air group *n* = 4) were loaded at 20 μg measured with Bradford protein assay (Bio-Rad, Hercules, CA, United States). Proteins were separated on a 4–12% Nu-Page Bis-Tris gel in MOPS buffer (Invitrogen, Carlsbad, CA, United States) at 140 V for 2 h followed by transfer to a PVDF membrane (Invitrogen) at 30 V for 1 h. The membrane was blocked in 5% non-fat milk for 1.5 h. Samples were then immunoblotted overnight at 4°C (5% BSA in 1 × TBST) with antibodies specific to Fis1 (1:500; Abcam, #ab71498), Mfn2 (1:500; Cell Signaling Technology, #9482), Drp1 (1:200; Santa Cruz Biotechnology, #sc-271583, Dallas, TX, United States), OPA1(1:200; Santa Cruz Biotechnology, #sc-393296, Dallas, TX, United States), Glyceraldehyde 3-phosphate dehydrogenase (GAPDH) (1:2000; #MAB374, Millipore). Following three 10-min washes (1 × TBST), immunoblots were incubated (5% BSA) for 1 h at room temperature with respective anti-rabbit and anti-mouse secondary antibodies (1:1000, Millipore). Blots were visualized with the Radiance Q Chemiluminescent Substrate (Azure Biosystems, Dublin, CA, United States), developed on an Azure 300 Chemiluminescent Western Blot Imaging System (Azure Biosystems, Dublin, CA, United States), and band optical density quantification was performed using NIH ImageJ software.

### Statistical Analysis

All data are expressed as mean ± SEM (standard error of the mean). The two-tailed Student’s *t*-test was used to compare the difference between the two groups. Statistical significance was set at *P* < 0.05. All statistical calculations were performed using GraphPad Prism 7 (La Jolla, CA, United States).

## Results

### CIA Exposure Altered Mitochondrial Morphology in ACC

**FIGURE 1:**
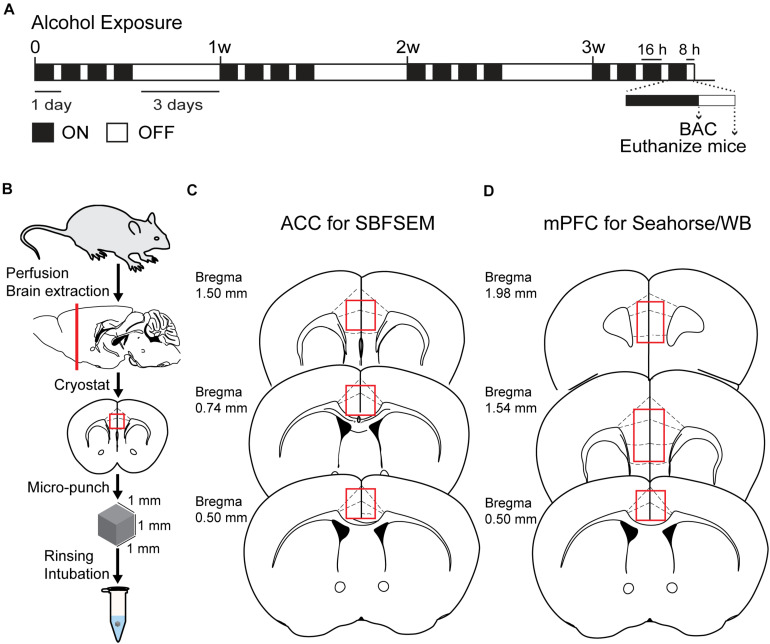
Chronic intermittent exposure to alcohol procedure and BAC. **(A)** Illustration of chronic intermittent alcohol exposure. **(B)** Process of tissue extraction for SBESEM study. **(C)** The red square displays ACC used in the SBFSEM study. **(D)** The red rectangle displays mPFC used in the Seahorse and WB studies.

**FIGURE 2:**
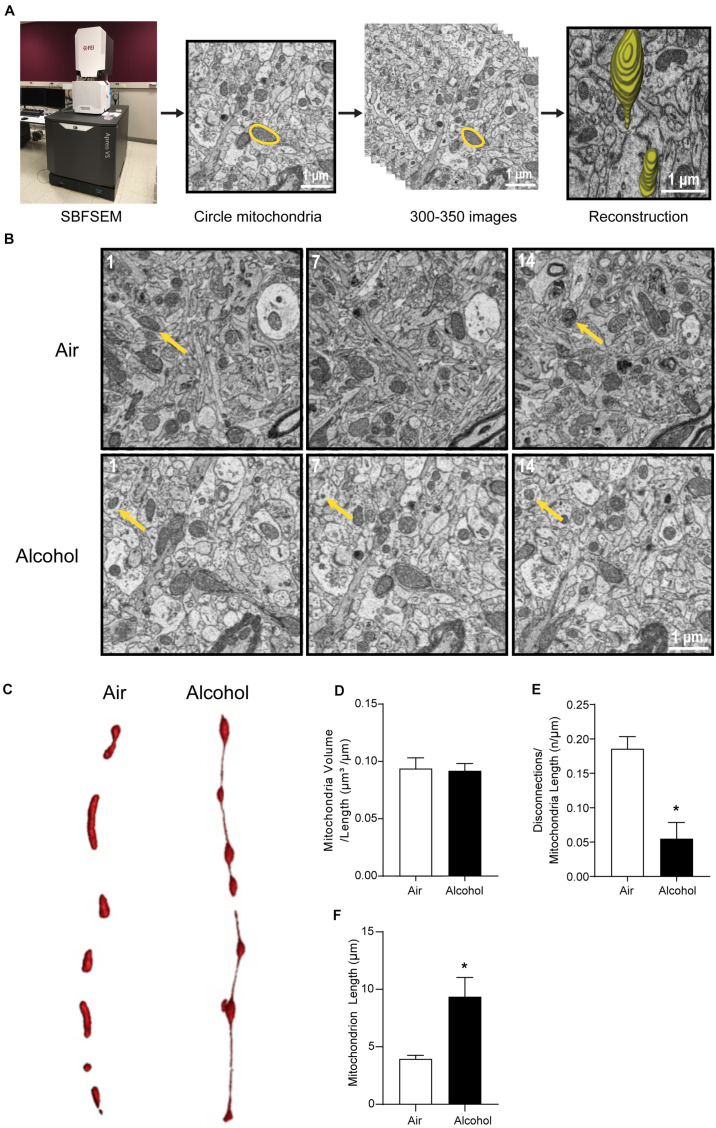
Three-dimensional morphological changes of the ACC mitochondria. **(A)** Representative patterning of mitochondria reconstruction in three-dimensional SBFSEM study.*: Mayo Clinic Microscopy and Cell Analysis Core’s FEI Apreo VS SEM. **(B)** A series of 50-nm sections showing representative mitochondria in ACC from the alcohol and air groups. The 1st, 7th, and 14th sections were selected from the same sample in two groups. Yellow arrows indicate representative mitochondria in those sections. **(C)** Representative three-dimensional mitochondrial reconstruction in air and alcohol groups. **(D)** Quantification of mitochondria volume. **(E)** Quantification of disconnections (mitochondrial ends) per mitochondrial length. **(F)** Quantification of mitochondrial length. *N* = 8 mitochondria per mouse. Asterisks (*) represent significance exists between two groups (*P* < 0.05) after unpaired Student’s *t*-test.

The CIA exposure schedule is illustrated in Figure 1A. At the end of the CIA exposure, the mean BAC in the alcohol group (*n* = 20) was 219.72 mg/dl (Figure 1B). The localization of ACC extracted for SBFSEM and mPFC extracted for Seahorse and western blotting were displayed in Figures 1C,D. We show the SBFSEM three-dimensional reconstruction patterning of dendritic mitochondria within ACC in Figure 2A. The mitochondrial connectivity patterning in the alcohol and air groups was observed and tracked in 300–350 continuing sections per mitochondrion (Figures 2A,B). MOAS was observed after reconstruction in the alcohol group with teardrop-shaped mitochondria (0.5 μm in diameter) connected by a thin double membrane up to 5 μm long (Figure 2C). Interestingly, mitochondrial reconstructions of air and alcohol-treated mice (two scans for each mouse) were similar in overall volume [unpaired Student’s *t*-test, *t*(12) = 0.1702, *P* > 0.05] (Figure 2D). However, the alcohol-treated mice displayed increased connectivity between mitochondria compared to air mice [unpaired Student’s *t*-test, *t*(12) = 4.506, *P* < 0.05] (Figure 2E). The average length of mitochondria in the alcohol group was 9.355 ± 1.67 μm compared with 3.938 ± 0.3161 μm in the air group. The mitochondrial average length was significantly higher in the alcohol group than in the air group [unpaired Student’s *t*-test, *t*(12) = 3.677, *P* < 0.05] (Figure 2F).

### Repeated CIA Diminishes Mitochondrial Respiration Capacity in mPFC of Mice

**FIGURE 3:**
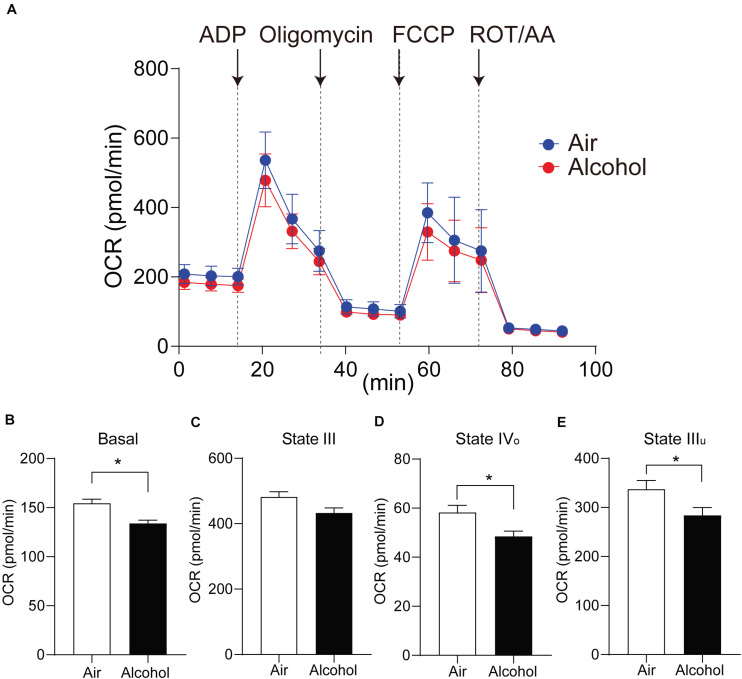
Mitochondria respiratory capacity changes in the mPFC from chronic intermittent alcohol exposure. **(A)** The oxygen consumption rate of isolated mitochondria in mouse mPFC. States changed accompanied by four injections of ADP and mito-stress drugs. **(B–E)** Comparison of mitochondrial OCR values in different states between the alcohol and air groups. Asterisks (*) represent significance exists between the two groups (*P* < 0.05) after unpaired Student’s *t*-test.

Isolated mitochondria from mPFC in the air and alcohol groups were immediately collected and loaded on the Seahorse XF96 Analyzer cell culture plate. Along with sequential injections of 20 μl 40 mM ADP, 22 μl 20 μM oligomycin, 25 μl 10 μM FCCP, and 27 μl 10 μM rotenone/antimycin A into 180 μl mitochondria-loaded MAS (final concentrations were 4 mM, 2 μM, 1 μM, and 1 μM, respectively), OCR and ECAR in each well were recorded every 6 min for 15 time points (Figure 3A). After a comparison between the alcohol group and the air group, significant differences were detected in basal level [unpaired Student’s *t*-test, *t*(9) = 2.584, *P* < 0.05], State IIIu [unpaired Student’s *t*-test, *t*(9) = 2.373, *P* < 0.05], and State IVo phases [unpaired Student’s *t*-test, *t*(9) = 2.796, *P* < 0.05] (Figures 3B–E), indicating baseline respiration, phosphorylating respiration, and MRC were inhibited in the alcohol group compared with the control group. The OCR after ADP supplement (State III) showed no significant change [unpaired Student’s *t*-test, *t*(9) = 1.958, *P* > 0.05].

### Fission and Fusion Protein Changes in mPFC of CIA-Treated Mice

**FIGURE 4:**
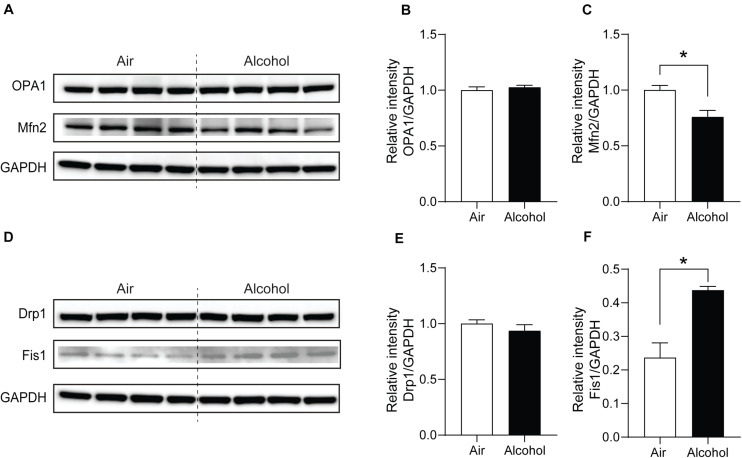
Mitochondria fission and fusion protein level changes in the mPFC from chronic intermittent alcohol treatment. Western blotting bands of **(A)** fusion proteins (OPA1 and Mfn2) and **(D)** fission proteins (Fis1 and Drp1) in the alcohol and air groups. The relative intensity of **(B,C)** fusion proteins (OPA1 and Mfn2) and **(E,F)** fission proteins (Fis1 and Drp1). Asterisks (*) represent significance exists between the alcohol and air groups (*P* < 0.05) after unpaired Student’s *t*-test.

Next, we explored the fission and fusion protein levels in the mPFC from the air and alcohol groups using western blotting. To compare protein expressions between air control (Air) and alcohol-exposed samples (Alcohol) directly, we loaded four samples in the middle of the gel. Since OPA1 (∼100 kDa) and Mfn2 (∼86 kDa) have similar molecular weights, we first used anti-Mfn2 antibodies. After stripping the bands, we used the ant-OPA1 antibodies, followed by GAPDH (36 kDa). For Drp1 (∼78 kDa) and Fis1 (∼17 kDa), we cut the blot and independently proceeded with the immunoblotting. We used GAPDH after stripping the blot using the anti-Drp1 antibodies. Mfn2, one of the fusion proteins, significantly decreased in the alcohol group [unpaired Student’s *t*-test, *t*(6) = 3.331, *P* < 0.05] (Figures 4A,C). Fission protein Fis1 (Figures 4D,F) was significantly increased in the alcohol group compared with the air control group [unpaired Student’s *t*-test, *t*(6) = 4.333, *P* < 0.05]. However, OPA1 did not show significant changes after alcohol treatment [unpaired Student’s *t*-test, *t*(6) = 0.6933, *P* > 0.05] (Figures 4A,B). Drp1 did not exhibit significance either [unpaired Student’s *t*-test, *t*(6) = 0.9853, *P* > 0.05] (Figures 4D,E). With three additional samples from each group, we confirmed the decreased Mfn2 and increased Fis1 in the alcohol group compared to the air control group (Supplementary Figure S1).

## Discussion

Our results suggest that CIA impairs mitochondrial morphology and function in the mPFC. The three-dimensional SBFSEM study of mitochondria from mouse ACC showed elongated mitochondria in mice exposed to chronic alcohol. To our knowledge, this was the first time that we observed an aberrant string form of mitochondria called MOAS in mice exposed to chronic alcohol. In addition, we presented the chronic alcohol exposure–inhibited aerobic respiration of isolated mPFC mitochondria. Our results suggest that increased mitochondrial fission (Fis1) and decreased fusion protein (Mfn2) are associated with the structural and functional changes of mPFC mitochondria.

It was known previously that alcohol increases mitochondrial fission in a concentration-dependent manner via OPA1 proteolytic change and Drp1 translocation (Bonet-Ponce et al., 2015). Drp1 inhibition dampens the permeability transition pore (mPTP) transiently and prohibits the release of reactive oxygen species (ROS) (Zhang et al., 2017). During the fission process, Drp1 cycles to the outer mitochondria membrane at scission sites and forms large homomultimeric complexes that circle mitochondrion in spirals (Ingerman et al., 2005). Fis1 anchors to the mitochondrial outer membrane via a transmembrane domain in its C-terminus and serves as the adaptor protein for dynamin proteins (Mozdy et al., 2000). After the recognition and Drp1 cycling, membrane fission occurs in an energy-dependent manner (Scott and Youle, 2010). Fission could facilitate the removal of damaged organelles via mitoautophagy (Westermann, 2012). Controversially, Fis1 is up-regulated to prohibit cell apoptosis by preventing ROS release after alcohol application in yeast (Kitagaki et al., 2007). Interestingly, after fission, the mitochondria with low membrane potential are less likely to re-fuse with the mitochondrial network in the absence of OPA1, which is known to maintain the equilibrium between mitochondrial fusion and fission (Herlan et al., 2003; Twig et al., 2008). Mfn2 is another outer membrane fusion protein that manages calcium uptake and metabolism with endoplasmic reticulum to sustain normal functions of mitochondria (De Brito and Scorrano, 2008). The cytosolic portion of Mfn2 contains guanosine triphosphate hydrolase enzymes along with two coiled-coil protein-interaction domains that mediate homotypic bindings between neighboring mitochondria (Koshiba et al., 2004). The down-regulation of Mfn2 may augment the pacing-induced Ca^2+^ transients, preventing Ca^2+^ accumulation in mitochondria to alleviate alcohol-induced oxidative stress (Chen et al., 2012).

According to previous *in vitro* studies, during the process of fragmentation induced by alcohol treatment, both Fis1 and Drp1 are increased (Kitagaki et al., 2007; Bonet-Ponce et al., 2015). In our study, we found no significant differences in Drp1 in mPFC between the alcohol and air groups, suggesting the total Drp1 in the mPFC does not change after alcohol treatment. Further investigations utilizing phosphorylated Drp1 primary antibody or mitochondria fractions may provide a better understanding of fission and fusion protein change in AUDs. Additionally, it is not clear whether the relative changes of Fis1 and Mfn2 protein expression in the mPFC of alcohol-treated mice are adaptations to the alcohol-induced mitochondrial respiratory deficits, yielding investigations of the relationship between mitochondrial respiration capacity and fission/fusion proteins. Overall, the increased Fis1 and decreased Mfn2 might facilitate the process of fragmentation, mitophagy, and apoptosis to alleviate oxidative stress induced by alcohol treatment.

Furthermore, our three-dimensional SBFSEM data suggest that mitochondrial connectivity is increased in the ACC of mice from the alcohol group with an MOAS phenomenon. Alcohol generates ROS and nitric oxide via induction of nicotinamide adenine dinucleotide phosphate (NADPH)/xanthine oxidase (NOX/XOX) and nitric oxide synthase in human neurons, indicating oxidative neuronal injury may lead to alcohol-induced neurodegeneration (Haorah et al., 2008). Of note, MOAS is found in mouse models of Alzheimer’s disease (AD) and postmortem tissues from human AD patients (Zhang et al., 2016), suggesting that alcohol-induced MAOS may be related to AD. MOAS was also observed in wild-type mouse brains that had experienced hypoxia or chronological aging, suggesting MOAS might be a compensatory adaption to bioenergetic stress to reserve mitochondrial function and prevent mitophagy (Zhang et al., 2016). Thus, we anticipate the MOAS might function as a protective response to the increased oxidative stress in alcohol-treated mice with promoting the residual mitochondrial function maintaining energy level. MOAS might also act as a compensation factor for the augmented mitophagy and apoptosis in alcohol-treated mice. Due to the fact that MOAS has been elucidated in neurodegenerative diseases like AD (Tyumentsev et al., 2018), alcohol effects on mitochondria might correlate with the development of neurodegenerative disorders. Theoretically, increased oxidative stress after CIA exposure might contribute to the overexpression of Fis1 in mouse mPFC to enhance mitochondria fission as a result of oxidative stress irritation. However, we observed MOAS formation in the ACC in the alcohol-treated group, which induces fission arrest and makes an adaptation to restitute energy supplement.

During fission in the process of apoptosis, fragmented mitochondria can release cytochrome c or other intermembrane proteins to impact neighboring mitochondria (Detmer and Chan, 2007). Although the relationship between mitochondrial dynamics and bioenergetics was previously demonstrated (Westermann, 2012), the causal relationship remained unknown. Proteomic analysis showed that 40 mitochondrial proteins, including key enzymes in β-oxidation, the tricarboxylic acid cycle, and amino acid metabolism, were altered after chronic alcohol consumption (Venkatraman et al., 2004). Deficits in complex I, III, IV, and V and 13 mitochondria-encoded polypeptides and redox centers were reported in isolated hepatic mitochondria of chronic alcohol-treated rats and contributed to compromises in the oxidative phosphorylation system (Coleman and Cunningham, 1991). Mitochondria accelerates the release of ROS and reactive nitrogen species in responding to alcohol consumption, which further enhances the oxidative injury of mitochondrial proteins and leads to mitochondrial dysfunction (Mantena et al., 2007). A previous study has shown that repeated ethanol withdrawal in rats significantly increased histone acetylation, accompanied by suppressed mitochondria respiration. Meanwhile, withdrawal-induced respiratory suppression can be exacerbated by trichostatin A, a histone acetylation promoter, further indicating that histone acetylation may dampen mitochondrial respiration capacity during ethanol withdrawal (Jung and Metzger, 2015). Alcohol withdrawal also inhibits mitochondrial complexes I–III, II–III, and IV, along with changes in synaptosomes (Karadayian et al., 2019). However, this study utilized whole cerebral cortices without an emphasis on a specific brain region. According to our Seahorse data, mitochondria in mPFC experienced low respiratory activity after consecutive alcohol treatment. The basal respiratory capacity was lower in the alcohol group compared with the control group despite the same mitochondria input. Non-phosphorylating respiration capacity (State IVo) also decreased in the alcohol group compared with the air control group. After FCCP activates ATP-uncoupled respiration (State IIIu) via freeing the oxidative phosphorylation chain from the proton motive force, mitochondria reach MRC without the assistance of ATP-synthase. In our study, the MRC was lower in the alcohol group, representing potential deficits in substrate oxidation. Due to the utilization of complex I-associated substrates, including malate and pyruvate, we further postulate alcohol might trigger complex I and/or complex IV deficits in the mPFC mitochondria. Interestingly, alcohol exposure did not alter ADP-dependent respiration (State III) but decayed the MRC (State IIIu). A compensation of oxidative phosphorylation induced by ATP synthase might exist to sustain state III and facilitate energy supplement after alcohol treatment, which might be the foundation of MOAS formation. In summary, CIA exposure dampened respiration capacity of the isolated mitochondria from the mPFC, and these mitochondrial deficits might be correlated with complex deficits induced by increased oxidative stress.

Previous findings showed prolonged alcohol drinking produces altered plasticity of intrinsic excitability with aberrant action potential firing and impaired after-hyperpolarization amplitude (Cannady et al., 2018). Kroener et al. (2012) reported that CIA-exposed mice displayed persistent alterations of *N*-methyl-D-aspartate receptor (NMDAR) mediated spike-timing-dependent plasticity in deep-layer neurons of the mPFC, the increase in the density of mushroom spines in basal dendrites from layer V neurons, and deficits in cognitive flexibility. Generally, mitochondrial activity inhibition leads to depressed synaptic transmission (Tang and Zucker, 1997). For example, rotenone, the complex I inhibitor, significantly impairs long-term potential in rat hippocampal neurons (Kimura et al., 2012). Selective inhibition of mitochondrial dihydroorotate dehydrogenase suppresses firing rates of neurons by modulating calcium buffering and spare respiratory capacity of mitochondria (Styr et al., 2019). Consistently, pharmacological enhancement of mitochondria respiration promotes an increase in synaptic density (Li et al., 2004). However, the cellular activity during inhibition of mitochondrial respiration also depends on neuronal characteristics (Bergmann and Keller, 2004). Motoneurons in the brainstem and hypoglossal nucleus are sensitive to hypoxia and calcium load and display increased action potential during complex IV inhibition (Bergmann and Keller, 2004). Chemical hypoxia induced by cyanide not only activates K^+^ channels but also increases a Na^+^ current and triggers depolarization of CA1 neuronal cells with more negative resting potentials (Englund et al., 2001). Antimycin-A, a complex III inhibitor, significantly increases GABAergic synaptic event frequency and affects miniature inhibitory postsynaptic current frequency by promoting generation of mitochondria-derived ROS (Accardi et al., 2014). Increased excitability of neurons has been shown previously in a CIA-exposed model (Pleil et al., 2015); however, the CIA impacts on pyramidal neuron activity in mPFC are still controversial (Tu et al., 2007; Weitlauf and Woodward, 2008). Further investigations are necessary to demonstrate the relationship between altered neuronal plasticity and mitochondria dysfunction in the mPFC after CIA exposure.

Chronic intermittent alcohol exposure also increases mPFC-associated maladaptive impulse and reward-seeking behaviors (Starski et al., 2019a). More specifically, the infralimbic and prelimbic cortex are involved in ethanol seeking, Pavlovian fear conditioning, and learning alternatives (Palombo et al., 2017; Mukherjee and Caroni, 2019). In this study, we chose to examine the EM-based structural changes within the ACC using the SBFSEM study because it is known to be vulnerable to CIA exposure, and the structural change of the ACC is closely correlated to alcohol and substance addiction (Marinkovic et al., 2013; Cheetham et al., 2014). However, ACC tissue from a single mouse does not provide enough mitochondria for the Seahorse approach while mPFC holds sufficient backup. mPFC was also utilized in western blotting due to its higher energy demand and more mitochondrial dynamics compared with other cortical areas (Arnsten et al., 2012). Importantly, projections from mPFC to subcortical regions display abnormalities in AUDs (Klenowski, 2018). A short application of alcohol reduces mPFC pyramidal neuronal activity and NMDAR-mediated post-synaptic currents (Tu et al., 2007; Weitlauf and Woodward, 2008). Conversely, the CIA procedure has been demonstrated to enhance excitatory post-synaptic currents in mPFC neurons (Kroener et al., 2012). Meanwhile, prolonged alcohol consumption induces a significantly greater level of dendritic restructuring in mPFC pyramidal neurons (Holmes et al., 2012; Kim et al., 2015). Therefore, we performed Seahorse and western blotting approaches using the mPFC tissue.

Several limitations of the current study should be noted. First, we utilized the forced involuntary alcohol exposure paradigm. Thus, our findings regarding the correlation between voluntary alcohol drinking and mitochondrial function are limited. Second, we have not presented comprehensive molecular experiments utilizing mitochondrial fractions. Third, we did not evaluate the multiple time points for alcohol withdrawal, especially a protracted withdrawal. Further study with larger sample sizes, a voluntary drinking paradigm, and comprehensive molecular studies with multiple time-point analysis will clarify the causal relationship between chronic alcohol drinking and aberrant mitochondrial morphology and function. Nonetheless, recognizing MOAS formation and mitochondrial functional inhibition in alcohol-treated mice builds a bridge to correlate alcoholism with neurodegenerative disorders and further emphasizes the detrimental effects of alcohol on the CNS. We also provided a novel perspective to correlate alcohol-induced AUDs with mitochondrial morphology alteration and functional prohibition in the mPFC.

## Data Availability Statement

Publicly available datasets were analyzed in this study. The data will be made available upon request.

## Ethics Statement

The animal study was reviewed and approved by Mayo Clinic Institutional Animal Care and Use Committee.

## Author Contributions

PSh and D-SC designed the research. PSh, DL, SC, and PSt performed the research. PSh and D-SC analyzed the data. PSh, LP, S-IH, and D-SC wrote the manuscript. All authors contributed to the article and approved the submitted version.

## Conflict of Interest

D-SC is a scientific advisory board member to Peptron Inc., and the Peptron had no role in the preparation, review, or approval of the manuscript; nor the decision to submit the manuscript for publication. The remaining authors declare that the research was conducted in the absence of any commercial or financial relationships that could be construed as a potential conflict of interest.

## Supplementary Material

Supplementary Figure 1 Mitochondria fission and fusion protein level changes in the mPFC from chronic intermittent alcohol treatment. **(A)** Western blotting bands of fusion proteins (OPA1 and Mfn2) and fission proteins (Fis1 and Drp1) in the alcohol and air group. **(B–E)** The relative intensity of fusion proteins (OPA1 and Mfn2) and fission proteins (Fis1 and Drp1). Mfn2 decreased significantly in the alcohol group compared with the air control group [unpaired Student’s *t*-test, *t*(4) = 3.742, *P* < 0.05]. Fis1 increased significantly in the alcohol group compared with the air control group [unpaired Student’s *t*-test, *t*(4) = 3.210, *P* < 0.05]. OPA1 did not show significant changes after alcohol treatment [unpaired Student’s *t*-test, *t*(4) = 0.4199, *P* > 0.05]. Drp1 also did not exhibit significance [unpaired Student’s *t*-test, *t*(4) = 0.6962, *P* > 0.05]. Asterisks (^∗^) represent significance exists between the alcohol and air groups *P* < 0.05) after unpaired Student’s *t*-test.
