# Supplementary figures and images for: Chronic Alcohol Exposure Induces Aberrant Mitochondrial Morphology and Inhibits Respiratory Capacity in the Medial Prefrontal Cortex of Mice
Source: Front Neurosci. 2020 Oct 22;14:561173. doi: 10.3389/fnins.2020.561173 (PMC7646256; doi:10.3389/fnins.2020.561173)

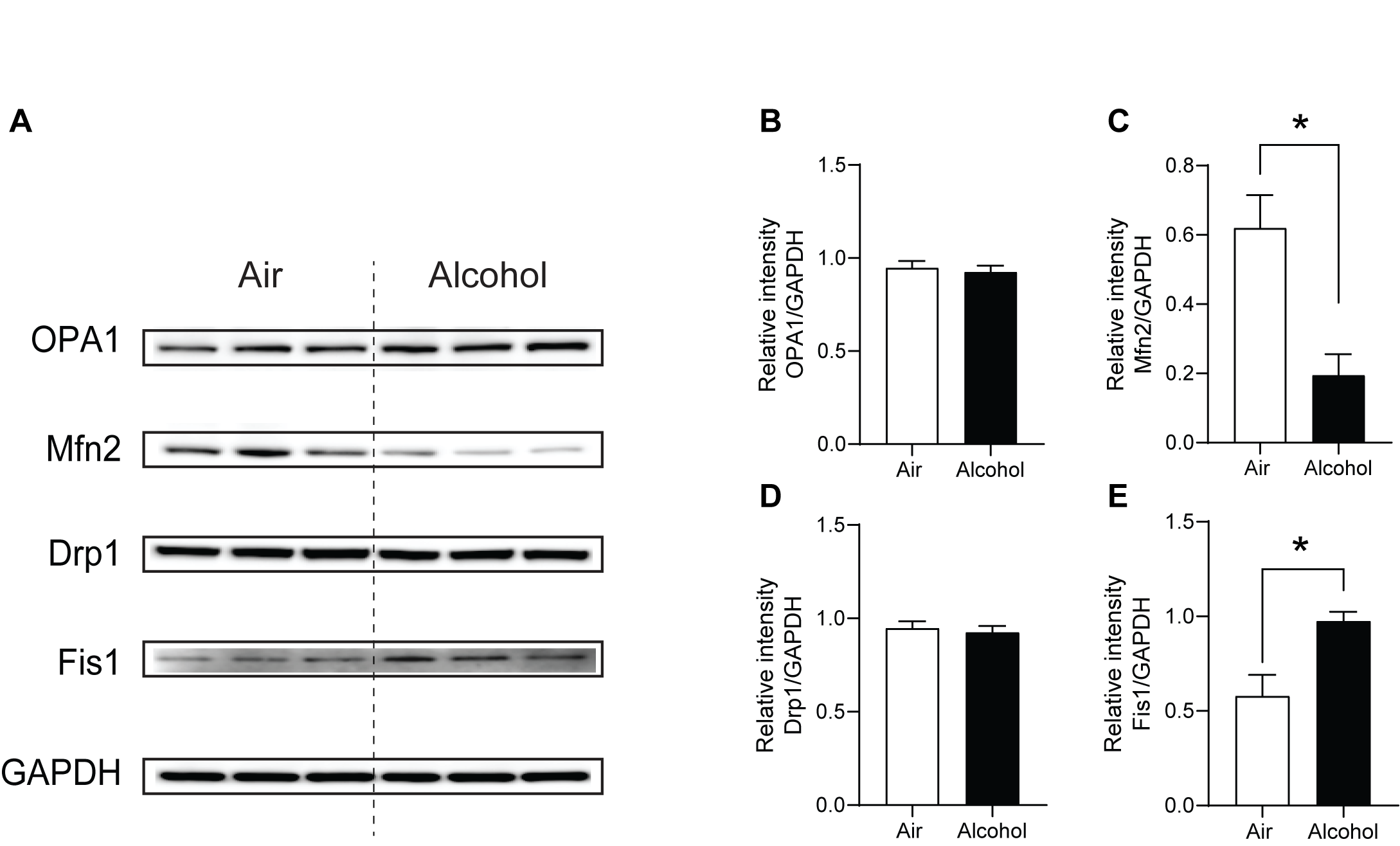

Supplement: Supplementary Figure 1 — Mitochondria fission and fusion protein level changes in the mPFC from chronic intermittent alcohol treatment. (A) Western blotting bands of fusion proteins (OPA1 and Mfn2) and fission proteins (Fis1 and Drp1) in the alcohol and air group. (B–E) The relative intensity of fusion proteins (OPA1 and Mfn2) and fission proteins (Fis1 and Drp1). Mfn2 decreased significantly in the alcohol group compared with the air control group [unpaired Student’s t-test, t(4) = 3.742, P < 0.05]. Fis1 increased significantly in the alcohol group compared with the air control group [unpaired Student’s t-test, t(4) = 3.210, P < 0.05]. OPA1 did not show significant changes after alcohol treatment [unpaired Student’s t-test, t(4) = 0.4199, P > 0.05]. Drp1 also did not exhibit significance [unpaired Student’s t-test, t(4) = 0.6962, P > 0.05]. Asterisks (∗) represent significance exists between the alcohol and air groups P < 0.05) after unpaired Student’s t-test. [file Image_1.TIF]
